# Role of hypoxia-related proteins in adenoid cystic carcinoma invasion

**DOI:** 10.1186/s13000-020-00967-3

**Published:** 2020-05-09

**Authors:** Raíssa Pinheiro de Mendonça, Giordanna Pereira Chemelo, Geovanni Pereira Mitre, Dimitra Castelo Branco, Natacha Malu Miranda da Costa, Fabrício Mesquita Tuji, Maria Sueli da Silva Kataoka, Ricardo Alves Mesquita, Sérgio de Melo Alves Júnior, João de Jesus Viana Pinheiro

**Affiliations:** 1 Department of Oral Pathology, School of Dentistry, Universidade Federal do Pará, Avenida Augusto Correa, 01, Belem, Para 66075-110 Brazil; 2 Multiprofessional Residency Program, Universidade Estadual do Pará, Rua do Una, 156, Belem, Para 66050-540 Brazil; 3 Department of Periodontology, School of Dentistry, Universidade de São Paulo, Avenida do Café, Subsetor Oeste, 11, Ribeirão Preto, São Paulo, 14040-904 Brazil; 4 Department of Oral Radiology, School of Dentistry, Universidade Federal do Pará, Avenida Augusto Correa, 01, Belem, Para 66075-110 Brazil; 5 Department of Oral Surgery and Pathology, School of Dentistry, Universidade Federal de Minas Gerais, Belo Horizonte, MG Brazil; 6 School of Dentistry, Cell Culture Laboratory, Universidade Federal do Pará (UFPA), Institute of Health Sciences, Avenida Augusto Correa, 01, Belem, PA 66075-110 Brazil

**Keywords:** Adenoid cystic carcinoma, Cell hypoxia, Invadopodia, Immunohistochemistry

## Abstract

**Background:**

Among cancers affecting the oral cavity, adenoid cystic carcinoma (ACC) is a relatively common malignant neoplasm. It has high rates of metastasis and recurrence and is associated with significant morbidity. During the progression of ACC, the oxygen concentration is reduced in specific areas of the tumour microenvironment, leading to intratumoural hypoxia. The expression of NOTCH1, a disintegrin and metalloproteinase 12 (ADAM-12), hypoxia-inducible factor 1 alpha (HIF-1α), and heparin-binding epidermal growth factor (HB-EGF) under hypoxic conditions has been implicated in invadopodia formation, tumour invasiveness, and metastasis. The aim of this study was to analyse the expression of these proteins to elucidate the mechanisms underlying ACC invasiveness.

**Methods:**

Fifteen ACC samples and 10 normal-looking salivary gland (SG) samples were used to investigate the expression of these proteins by immunohistochemistry. Primary antibodies against NOTCH1, ADAM-12, HIF-1α, and HB-EGF were used.

**Results:**

The immunoexpression of all proteins was higher in ACC samples than in SG samples (*p* < 0.05).

**Conclusions:**

There was increased expression of proteins associated with hypoxia and tumour invasiveness in ACC samples, which indicates a possible role of these proteins in the biological behaviour of this tumour.

## Background

Adenoid cystic carcinoma (ACC) is a relatively common malignant neoplasm that mainly affects the minor salivary glands in various regions, including the palate. It accounts for approximately 12–27% of all cases of salivary gland tumours. The biological behaviour of ACC is characterised by slow and highly invasive growth, which has been the subject of many studies; most of these studies have focused on the high rates of metastasis and recurrence and the significant morbidity [1–3].

The invasion of ACC depends on the ability of tumour cells to remodel and degrade the extracellular matrix (ECM). Studies have suggested that localised proteolysis might be mediated by invadopodia, which are finger-like cellular protrusions with intrinsic proteolytic activity that have been linked to the initiation of tumour invasion and the activation of matrix metalloproteinases (MMPs) [4–6]. Recently, it was observed that oxygen-poor microenvironments favour the formation and activity of invadopodia [7–9].

During tumour progression, the oxygen concentration in the tumour microenvironment decreases, causing intratumoural hypoxia [9]. This process triggers several biochemical responses that can result in a series of compensatory cellular mechanisms that allow neoplastic progressions, such as cell invasion, metastasis, and the activation of certain proteins and transcription factors [10]. Clinically, hypoxia is related to poor prognosis and reduced survival rates, especially in cases of head and neck tumours [11].

Some proteins and transcription factors have been directly implicated in hypoxia-induced paracrine and autocrine signalling pathways, including hypoxia-inducible factor 1 (HIF-1), a transcription factor that regulates hypoxia-responsive elements [12]. Activation of HIF-1α plays an essential role in the invasive process, as it regulates specific genes involved in cell motility, adhesion and invasion through invadopodia, which are critical for tumour growth and aggressiveness [9, 13]. Overexpression of HIF-1α has been detected in several human tumours [14]. Thus, the relationship between hypoxia, HIF-1α, and invadopodia formation clearly influences tumour aggressiveness.

Additional proteins are related to the hypoxia-induced formation of invadopodia, such as a disintegrin and metalloproteinase 12 (ADAM-12). The literature on the association between ADAM-12 and some malignant tumours, such as breast [15] and prostate cancer [16], is vast, indicating its direct relationship with the pathophysiology of these neoplasms. ADAM-12 exhibits catalytic activity by cleaving certain ligands that are biologically important for tumour growth, such as tumour necrosis factor-alpha (TNF-α), epidermal growth factor (EGF), and transforming growth factor-alpha (TGF-α) [17, 18]. Díaz et al. observed that ADAM-12 and HIF-1α are part of the same molecular mechanism responsible for increasing tumour aggressiveness in a hypoxic microenvironment [7].

Another growth factor that may contribute to the increased aggressiveness of ACC in response to hypoxia is the heparin-binding epidermal growth factor (HB-EGF). HB-EGF is part of the EGF family and can bind to EGFR and ErbB4, which have been associated with malignant transformation in several human tumours [19]. HB-EGF is synthesised as pro-HB-EGF, which is subsequently cleaved by ADAM-12 to release sHB-EGF (soluble form of HB-EGF), which promotes invadopodia formation [7, 20].

In this context, another crucial protein that participates in invadopodia formation is NOTCH1. NOTCH signalling can both promote and restrict cell differentiation, largely dependent on the microenvironment and crosstalk with other signalling pathways [21]. Notch signalling is initiated when its ligands interact with the receptor, and this pathway ultimately induces the transcription of several protein-coding genes, including the one encoding ADAM-12 [22]. NOTCH1 is directly related to the invasive nature of certain malignancies [23] and is necessary to enhance invadopodia formation and, consequently, promote cell invasion [7]. Under hypoxic conditions, the NOTCH signalling pathway can be activated to trigger the HIF-1α transcription factor, thus, enabling the stabilisation of the intracellular response [24].

The tumour microenvironment is an important regulator of invasive behaviour; therefore, characterising this environment might reveal associated molecular mechanisms. This knowledge could be used to improve the ACC diagnosis and treatment approaches. Thus, the aim of this study was to analyse the immunoexpression of NOTCH1, HIF-1α, ADAM-12, and HB-EGF in ACC to clarify the initial steps in the invasion cascade and their relationship with hypoxia.

## Methods

### Samples

The Human Research Ethics Committee of the Health Sciences Institute of the Federal University of Pará approved this research (protocol 1903223). Fifteen ACC from minor salivary glands and 10 normal-looking salivary gland (SG) samples were retrieved from the repository of the Department of Oral Pathology, School of Dentistry, Centro Universitário do Pará (CESUPA), Belem, PA, Brazil. Clinical data were obtained from 11 cases of ACC.

### Immunohistochemistry

Formalin-fixed, paraffin-embedded tissues were evaluated by immunohistochemistry. Sections (3-μm thick) were mounted on 3-aminopropyltriethoxysilane-coated slides (Sigma Chemical Corp, St. Louis, MO, USA), dewaxed in xylene and hydrated in a graded ethanol series. Antigen retrieval was performed for 30 s with a citrate buffer (pH 6.0) in a Pascal chamber (Dako, Carpinteria, CA, USA). Sections were immersed in 3% H_2_O_2_ in methanol for 20 min to inhibit endogenous peroxidase activity and then blocked with 1% bovine serum albumin (BSA, Sigma®) in phosphate-buffered saline (PBS) for 1 h. Slides were incubated with primary antibodies against NOTCH1 (1:250, Rabbit Anti-NOTCH 1 Intracellular Polyclonal Antibody, Millipore, Temecula, CA, USA), HIF-1α (1:50, Rabbit Anti-HIF-1α Monoclonal Antibody, Millipore®), ADAM-12 (1:15, Rabbit Anti-ADAM 12 Polyclonal Antibody, BIOSS, Boston, MA, USA) and HB-EGF (1:30, Mouse Anti-HB-EGF Monoclonal, R&D Systems, Minneapolis, MN, USA). All primary antibodies were diluted in PBS and incubated with the slides for 1 h at room temperature. Afterwards, the sections were incubated for 40 min with a labelled streptavidin/biotin HRP detection system (Dako®). Diaminobenzidine (Dako®) was used as the chromogen, and sections were counterstained with Mayer’s haematoxylin (Sigma®). For negative controls, primary antibodies were replaced with non-immune serum.

### Immunostaining evaluation

To assess the intensity of NOTCH1, HIF-1α, ADAM-12, and HB-EGF staining, brightfield images from at least five randomly selected fields in each sample were acquired using an Axio Scope microscope (Carl Zeiss, Germany) equipped with a CCD colour camera (Axiocam HRc; Carl Zeiss). Images were acquired at the same magnification (40x). The TIFF images underwent colour deconvolution (plug-in written by Gabriel Landini, http://www.dentistry.bham.ac.uk/landinig/software/software.html) of ImageJ (public domain software developed by Wayne Rasband; NIMH, NIH, Bethesda, MD, USA, http://rsbweb.nih.gov/ij/) to separate automatically the diaminobenzidine (DAB) colour from the haematoxylin and residual complementary colour. After image segmentation, the area and percentage of total staining were quantified. The differences between ACC and SG, neoplastic cells and stromal cells were included in the morphometry analysis.

### Statistical analysis

Data were analysed using GraphPad Prism 6 software (Graph Pad Software, Inc., San Diego, CA, USA). The non-parametric Kruskal–Wallis test was used to evaluate the significance of differences in protein expression between the stroma and tumour parenchyma and between the ACC and control samples. Spearman correlation test and linear regression were also performed.

## Results

### Clinical and pathological data

Clinical data were available for only 11 of the 15 cases included in this study. The clinical and pathological data of patients with ACC are presented in Table 1. In the sample cohort, the mean age was 64.3 years, with 54.54% of individuals older than the mean and the remaining 45.45% younger than the mean. The female sex was more prevalent, accounting for 72.2% of the cases. None of the patients were smokers or consumed alcohol (100%). Four patients (36.36%) used prosthetics. The most prevalent sites of ACC included the palate (54.54%), upper lip, lower lip, nasopharynx, oropharynx, and mandible (9.09% each).

**Table 1:** Distribution of the 11 ACC cases according to demographic, lifestyle, and clinicopathological variables

Variable	Categories	n (%)
Age (years)	≤64.3	5 (45.45)
	> 64.3	6 (54.54)
Sex	Male	3 (27.27)
	Female	8 (72.2)
Race	Caucasian	2 (18.19)
	Non-Caucasian	9 (81.81)
Smoking habit	Yes	0 (0)
	No	11 (100)
Alcohol consumption	Yes	0 (0)
	No	11 (100)
Use of prosthesis	Yes	4 (36.36)
	No	7 (63.63)
Localisation	Palate	6 (54.54)
	Upper Lip	1 (9.09)
	Inferior Lip	1 (9.09)
	Nasopharynx	1 (9.09)
	Rhinopharynx	1 (9.09
	Mandible	1 (9.09)

### Higher expression of HIF-1α, NOTCH1, ADAM-12, and HB-EGF in ACC in comparison with normal-looking salivary gland samples

All ACC samples had higher protein expression of HIF-1α, NOTCH1, ADAM-12, and HB-EGF than the SG samples used as a control (Table 2). In addition, in ACC samples, the immunoexpression of these proteins was higher in the parenchyma of tumour cells than in the stromal cells (Table 3).

**Table 2:** The *p* values when comparing HIF-1α, NOTCH1, ADAM-12, and HB-EGF expression among ACC and SG, Kruskal–Wallis test

		HIF-1alfa (%)			NOTCH1 (%)			ADAM-12 (%)			HB-EGF (%)		
Lesion	n	mean	SD	p<	mean	SD	p<	mean	SD	p<	mean	SD	p<
ACC	15	56.31	14.36	0.05	65.63	11.06	0.01	61.67	10.51	0.05	71.48	14.49	0.001
SG	10	30.53	12.98		41.20	7.45		38.00	7.85		37.44	6.80	

*ACC* adenoid cystic carcinoma; *SG* salivary gland

**Table 3:** The p values when comparing HIF-1α, NOTCH1, ADAM-12, and HB-EGF expression among parenchyma and stroma, Kruskal–Wallis test

		HIF-1alfa (%)			NOTCH1 (%)			ADAM-12 (%)			HB-EGF (%)		
Tissue	n	mean	SD	p<	mean	SD	p<	mean	SD	p<	mean	SD	p<
PAR	15	56.31	14.36	0.01	65.63	11.06	0.001	61.67	10.51	0.01	71.48	14.49	0.001
STR	10	24.51	11.12		28.15	12.80		29.66	11.30		15.00	5.96	

*PAR* parenchyma; *STR* stroma

### HIF-1α, NOTCH1, ADAM-12, and HB-EGF Immunoexpression patterns

The immunoexpression of all proteins was categorised into two patterns with regard to localisation and intensity, according to Weber et al. [25]. The localisation of the immunostaining was classified as nuclear or cytoplasmic, and the intensity was classified as low (< 50% stained cells) or high (≥50% stained cells).

**Fig. 1:**
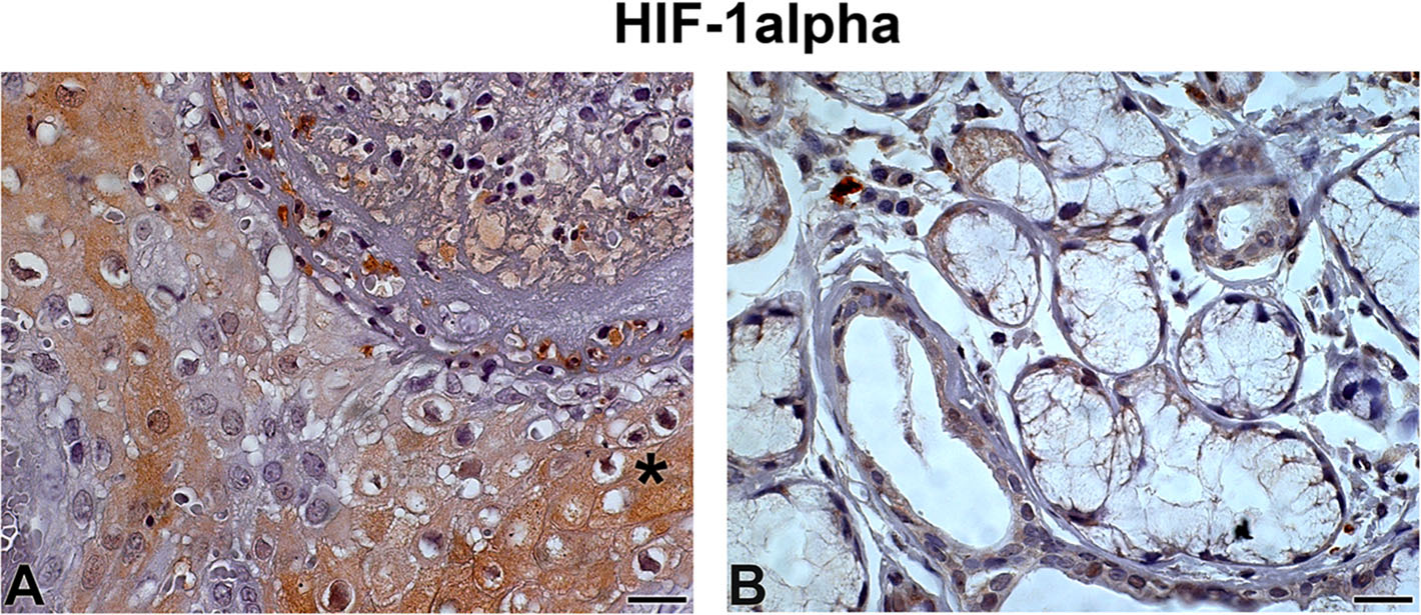
HIF-1α Legend: Immunostaining of hypoxia-inducible factor 1 alpha in adenoid cystic carcinoma (**a**) and normal-looking salivary gland (**b**). Immunoperoxidase. Scale bar: 20 μm

HIF-1α expression in the tumour parenchyma was high and was detected in both the nucleus and cytoplasm, whereas the stroma showed a low staining intensity (Fig. 1a). In addition, we observed strong staining of HIF-1α in perineural invasion areas (1a, asterisk) and necrotic areas. The SG samples had a low immunostaining intensity (Fig. 1b).

**Fig. 2:**
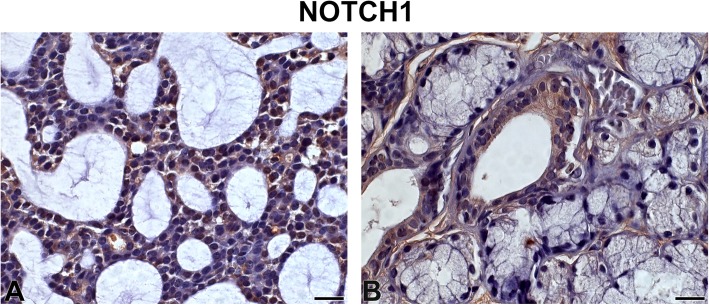
NOTCH1 Legend: Immunostaining of NOTCH1 in adenoid cystic carcinoma (**a**) and normal-looking salivary gland (**b**). Immunoperoxidase. Scale bar: 20 μm

NOTCH1 showed high-intensity immunostaining with localised distribution in the nucleus and cytoplasm of tumour parenchyma cells. The stromal immunoexpression was low and focal (Fig. 2a). SG samples showed low-intensity staining (Fig. 2b).

**Fig. 3:**
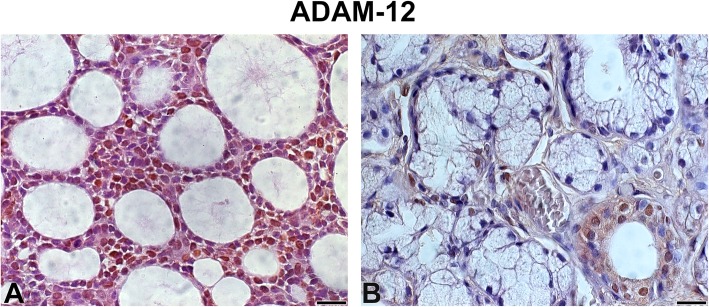
ADAM-12 Legend: Immunostaining of a disintegrin and metalloproteinase 12 in adenoid cystic carcinoma (**a**) and normal-looking salivary gland (**b**). Immunoperoxidase. Scale bar: 20 μm

There was high-intensity staining of ADAM-12 in the nucleus of tumour parenchyma cells, whereas the stroma showed low-intensity staining (Fig. 3a). The SG samples also showed low-intensity staining (Fig. 3b).

**Fig. 4:**
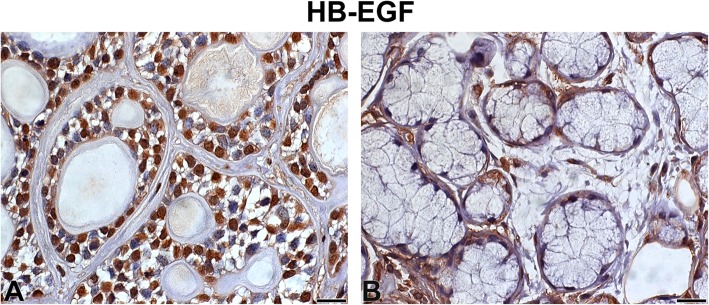
HB-EGF Legend: Immunostaining of heparin-binding epidermal growth factor in adenoid cystic carcinoma (**a**) and normal-looking salivary gland (**b**). Immunoperoxidase. Scale bar: 20 μm

The immunoexpression of HB-EGF was high and localised in the nucleus and cytoplasm of tumour parenchyma cells, whereas in the stroma, there was low-intensity immunostaining (Fig. 4a). The SG samples also showed low-intensity immunostaining (Fig. 4b).

### NOTCH1 showed correlation and association with HIF-1α and HB-EGF

The Spearman test showed a positive correlation between NOTCH1 and HIF-1α (r_s =_ 0.6814, *p* < 0.0026) and between NOTCH1 and HB-EGF (r_s =_ 0.6495, *p* < 0.0048) (Table 4). In addition, the linear regression shows an association between NOTCH1 and HIF-1α (*r*^2^_=_ 0.4211, p < 0.0048) and between NOTCH1 and HB-EGF (*r*^2^_=_ 0.3047, *p* < 0.0216) (Table 5).

**Table 4:** Positive Spearman’s rank correlation test of the labelling area of proteins in neoplastic cells of ACC

ACC neoplastic cells			
Protein 1	Protein 2	r_*s*_	*p* - value
NOTCH1	HIF-1α	0.6814	0.0026**
	HBEGF	0.6495	0.0048**

r_s_: Spearman’s coefficient of rank correlation; **p < 0.01.

**Table 5:** Linear regression showed association among NOTCH1 and HIF-1α and HBEGF of labelling area of proteins in neoplastic cells of ACC

ACC neoplastic cells			
Protein 1	Protein 2	r^2^	p - value
NOTCH1	HIF-1α	0.4211	0.0048**
	HBEGF	0.3047	0.0216*

*r*^2^: Coefficient of determination; *P < 0.05; **P < 0.01

## Discussion

In this study, we observed a higher expression of HIF-1α, NOTCH1, ADAM-12, and HB-EGF in ACC samples than in normal-looking salivary gland samples from healthy individuals.

Within the data, there was a mean age of 64 years and the female sex was more prevalent, similar to those previously reported in the literature [1–3, 26]. None of the patients were smokers or consumed alcohol, corroborating the fact that there are no distinct risk factors, and smoking is not known to affect incidence [26]. The most prevalent site of ACC samples was the palate, which was the most prevalent ACC site [3].

It was previously established that HIF overexpression allows cells to adapt to a hypoxic environment and proliferate, leading to increased invasion, metastasis, and tolerance to radiation and chemotherapy [27–29]. The expression of HIF-1α in ACC has been investigated, and in vitro studies have demonstrated that HIF-1α knockdown decreases cell proliferation, invasion, and migration, suggesting that this transcription factor might be a promising therapeutic target [30, 31]. In this context, our results demonstrated a high expression of NOTCH1, HIF-1α, ADAM-12, and HB-EGF, suggesting that these proteins contribute to ACC tumourigenesis.

During neoplastic progression, tumour cells generate mechanisms to survive the negative effects of hypoxia, one of which is the activation and stabilisation of the transcriptional activity of HIF-1α, which can then regulate the NOTCH1 signalling pathway [24].

Initially, HIF-1α is localised in the cytoplasm, where it binds to the HIF-1β subunit and then translocates to the nucleus, where it promotes the transcription of genes related to hypoxia [32]. The HIF-1α activity enables hypoxic tissue to adopt an aggressive phenotype characterised by angiogenesis and even metastasis, thus, contributing to tumour progression, metabolic changes, cell proliferation, and the activation of signalling pathways [33]. The ACC samples showed higher immunoexpression of HIF-1α compared to SG samples, indicating that NOTCH1 signalling was stabilised. These events trigger the transcription of several genes, including ADAM-12. Our study found higher expression of ADAM-12 in ACC, which may be associated with the release of mitogens in this neoplasm.

The ACC samples showed higher expression of NOTCH1 compared to control samples. When the extracellular domain of NOTCH1 is activated by one of its ligands (Jagged 2), the intracellular domain of NOTCH (DICN) is cleaved and translocated to the nucleus, where it binds to the CSL transcription factor [21]. This activity explains the nuclear and cytoplasmic localisation of NOTCH1 in the current study. NOTCH1 is key for invadopodia formation and therefore promotes cell invasion [7].

During tumour progression, growth factors are liberated through the activity of MMPs such as ADAM-12, a disintegrin responsible for ECM proteolysis [34]. ADAM-12 and other MMPs, such as 1, 2 and 9, may contribute to the release of mitogens, thereby increasing local aggressiveness and tumour morbidity [35]. Previous studies published by our research group have consistently shown that ADAM-12 can localise to the membrane, cytoplasm, and nucleus [36, 37], a finding that has been confirmed by other studies that reported the presence of other MMPs, including those in the ADAM family, in the nucleus [38]. In the current study, ADAM-12 was expressed in the nucleus of tumour parenchyma cells, which is probably associated with the high capacity of ACC for invasion and metastasis. High ADAM-12 expression in the nucleus may represent another signalling pathway that influences ACC progression, since the expression of ADAM-12 is also effected by NOTCH activation of the nuclear factor-κB (NF-κB) via a CSL-dependent mechanism. NF-κB activation represses the transcription of miRNA-29 (a suppressor of ADAM-12 transcription), which in turn upregulates the transcription of ADAM-12 [18, 35]. Some studies have noted that MMP family enzymes transcribed in the nucleus affect apoptosis and regulate cell proliferation and proteinases, among other phenomena [39, 40].

ADAM-12 is crucial for the release of certain growth factors, such as HB-EGF, as it facilitates binding to receptors, especially EGFR, leading to signals that stimulate cellular events such as proliferation, differentiation, and migration [20, 41]. ADAM-12 can, therefore, contribute to the cleavage of HB-EGF and thereby enhance HB-EGF immunoexpression. Due to its transmembrane location, Hieda et al. [42] believe that the cytoplasmic portion of HB-EGF translocates to the nucleus when cleaved. ProHB-EGF is mostly expressed on the cell surface; however, in the presence of the appropriate stimuli, it is cleaved and probably undergoes retrograde transport to the Golgi apparatus through endosome recycling. It is subsequently directed to the nucleus, where it diffuses or is actively transported before binding the nuclear membrane [42]. After pro-HB-EGF cleavage, the domain that translocates to the nucleus inhibits proteins that regulate and control the cell cycle, such as PLZF and Bc16. This phenomenon favours and promotes tumour invasiveness once the normal cell cycle is compromised [43, 44]. Our findings showed predominant HB-EGF immunostaining in the nucleus, suggesting that, in addition to inducing the formation of invadopodia, HB-EGF might also promote cell proliferation [45]. Therefore, tumours show increased capabilities for tissue invasion and growth and, thus, increased metastasis ability.

In this sense, studies have shown that, in hypoxic conditions, NOTCH1, HIF-1α, and HB-EGF act together for invadopodia formation [7]. In our study, we observed a positive correlation and association between these proteins (Tables 4 and 5), which may indicate possible crosstalk between them for invadopodia formation and consequently ACC invasion.

The results of the current study, together with those of other relevant reports in the literature, suggest that, under hypoxic conditions, HIF-1α levels are stabilised, and NOTCH1 signalling is activated by increasing the levels of JAG2, which recruits γ-secretase to cleave NOTCH1, releasing the intracellular domain that then translocates to the nucleus. This event triggers the transcription of ADAM-12, which then cleaves and induces the activation of the pro-HB-EGF domain, releasing HB-EGF into the extracellular space. The secreted HB-EGF binds to EGFR, leading to invadopodia formation in tumour cells in a hypoxic environment. This may further promote the formation of invadopodia in cells located in normoxic zones, suggesting crosstalk between areas of normoxia and hypoxia within a tumour [7]. In addition, we observed intense labelling of HIF-1α in areas of necrosis and perineural invasion. The labelling in necrotic areas was expected, as there is a direct relationship between hypoxia and necrosis [30, 31]. Interestingly, intense labelling of HIF-1α was found in the perineural invasion region, which may indicate a relationship between this signalling pathway and invadopodia formation that promotes perineural invasion. Some studies established a relationship between perineural invasion and HIF-1α in certain malignancies [46, 47], but to the best of our knowledge, our study is the first to identify this correlation in ACC.

## Conclusions

We suggest that NOTCH1, HIF-1α, ADAM-12, and HB-EGF are directly related to the mechanism of ACC invasion. These proteins are most likely components of the same cell signalling pathway during hypoxia that leads to enhanced invadopodia formation and, consequently, increased tumour invasiveness.

## Data Availability

All data generated or analysed during this study are included in this published article.

## Abbreviations

ACC: Adenoid cystic carcinoma
ADAM-12: A disintegrin and metalloproteinase 12
BSA: Bovine serum albumin
EGF: Epidermal growth factor
ECM: Extracellular matrix
HB-EGF: Heparin-binding epidermal growth factor
HIF-1: Hypoxia-inducible factor 1
HIF-1α: Hypoxia-inducible factor 1 alpha
MMPs: Matrix metalloproteinases
SG: Normal-looking salivary gland
PBS: Phosphate-buffered saline
TGF-α: Transforming growth factor-alpha
TNF-α: Tumour necrosis factor-alpha
